# Morphology and epidemiological study of idiopathic scoliosis among primary school students in Chaozhou, China

**DOI:** 10.1186/s12199-021-00989-3

**Published:** 2021-07-03

**Authors:** Zemin Cai, Ruibin Wu, Shukai Zheng, Zhaolong Qiu, Kusheng Wu

**Affiliations:** 1 Department of Preventive Medicine, Shantou University Medical College, No.22, Xinling Rd, Shantou, 515041 Guangdong Province China; 2 Chaozhou People’s Hospital, Chaozhou, 521000 Guangdong Province China

**Keywords:** Idiopathic scoliosis, Scoliosis screening, Prevalence, Associated factors

## Abstract

**Background:**

Idiopathic scoliosis (IS) affects patients’ quality of life, yet there have been few reports of its morphology and epidemiological study in the southeast region of China. The aim of this study is to access the curve characteristics, prevalence, and factors associated with IS in Chaozhou city.

**Methods:**

A cross-sectional study was performed in 2018, in which scoliosis screening was conducted among 5497 primary school students in Chaozhou city. Then, a case-control study based on the screening involving 2547 children was followed for the exploration of the associated factors. The questionnaires covering demographic characteristics, postural habits, cognition and self-sensation of scoliosis, and physical conditions were addressed for the investigation. *ORs* with 95%*CIs* were calculated based on logistic regression analysis to evaluate the factors associated with scoliosis.

**Results:**

The prevalence of IS among primary school students was 6.15% in Chaozhou city, with 4.04% for males and 8.71% for females. The average Cobb angle was 15° (range 8 to 37°). Multiple logistic regression analysis suggested that female (*OR*=2.45), BMI (*OR*=0.67), having myopia (*OR*=1.49), self-sensation of scoliosis with symptoms (*OR*=5.52), insufficient sleep time (*OR*=2.65, 3.33), and less exercise time (*OR*=7.09, 7.29) were significantly associated with IS.

**Conclusions:**

The prevalence of IS among primary school students in Chaozhou was at an average level, and it was significantly higher in females than in males. Lower body mass, having myopia, insufficient sleep time, and lower physical activity were associated with IS.

## Background

Scoliosis is a transverse and rotated spine curvature that usually occurs in children or around puberty [1]. Clinically, patients frequently suffer from back pain symptoms. For severe patients, scoliosis can affect limb symmetry, appearance, and even affect the functions of the heart, lung, gastrointestinal or other internal organs, motor coordination ability, or activity ability [2, 3]. According to the pathogenesis, scoliosis can be sorted as idiopathic scoliosis (IS) and non-idiopathic scoliosis. The proportion of IS is 80% among scoliosis, mainly in adolescent girls [4].

IS screening among school children is vital for diagnosis and confirming progression of the curve. According to the previous researche s[5–8], the IS prevalence was mostly between 2% and 16%. The prevalence of IS in females was about four times higher than that in males [9]. With a curve progression of ≤10° studied, the prevalence of scoliosis had no difference in gender. However, the prevalence became higher in females when the curve progressed.

Due to the complicated development of scoliosis, the pathophysiological mechanism is debatable. Some studies [10, 11] indicated that it is related to genetic factors, endocrine abnormalities, nervous system dysfunction, abnormal growth and development, and any other factors, while other studies [12, 13] suggested that IS was mainly related to exercise and habitual posture. Adolescence is a period of the rapid development of scoliosis, but it is also a critical period for recovery and correction [14]. Adolescent scoliosis is a common spinal disease in adolescents, which is asymptomatic in its early stages and surgery is often required when spinal deformity occurs. Early screening to detect scoliosis can effectively prevent disease progression [15]. Therefore, attention should be paid to this period to reduce the pathogenic factors to scoliosis. Early screening, diagnosis, and intervention are important for the phased treatment of scoliosis.

There have been few reports of scoliosis prevalence based on large-scale morphological and epidemiological surveys among primary school children in the southeast coastal region of China. The aim of this study is to access the curve characteristics, prevalence, and the factors associated with IS by scoliosis screening and investigation among primary school students from grade 1 to 6 in Chaozhou city (Teochew), so as to provide clues for prevention, intervention, and treatment of IS and promotion of spinal health.

## Methods

### Study design and subjects

This study was divided into two stages. In the first stage, a cross-sectional study was carried out to survey the morphology and prevalence of IS among primary school students (grades 1 to 6) in Chaozhou city based on a cluster random sampling method from October to November, 2018. Scoliosis screening for these primary school students was performed based on available international methods (introducing below in detail).

In the second stage, a case-control study based on the screening survey was conducted, which involved the screening out scoliosis students as cases, and the students without scoliosis from the same classes as controls, to explore the factors associated with IS.

The study was approved by the human ethics committee of Shantou University Medical College. All participates were noticed of the contents of this study and attached with their privacy and confidentiality commitments utilizing voluntary.

### Scoliosis screening

#### Screening criteria

Spine and chest deformities (including treatment with a brace), musculoskeletal anomalies, neurological disorders, and operation history were questioned and excluded from the study.

The screening method were (1) students were examined in an upright standing position to observe head lean, shoulder asymmetries, unequal inferior angle of the scapula, scapular prominence, waist asymmetries, or pelvis lean. (2) Adams’ forward bending test (FBT) was examined to observe thorax asymmetries, scapular asymmetries, waist asymmetries, pelvis lean, and spinous process line is not in the longitudinal midline. Students with at least one positive test were inspected angle of trunk rotation (ATR), which was screened by the MeshLab 3D (ISTI-CNR) [16]. Furthermore, the inclusion criteria were that a curve progression of ≥3°was used as ATR cut-off value and suspected of scoliosis. The standard Cobb method was used to measure X-ray examination, and cases with a Cobb angle of ≥10°were accepted as scoliosis.

#### Screening procedure

The sample size of the screening was calculated as follows: $n=\frac{{u_{\upalpha}}^2 pq}{d^2}$, where p is the prevalence of scoliosis among primary school students, and value as 6% according to literature report; d is the sensitivity level (margin error), d=0.15p. Finally, we regard 3068 as the minimum sample size after increasing 10%. The physical examination was performed by orthopedics and rehabilitation physicians to screen-positive patients. Then the first positive screening participants were recorded for the age, gender, class, ID number, and other information and taken the ATR as reference data. The suspected scoliosis students were performed an X-ray examination and were made a definite diagnosis according to the X-ray results.

### Investigation on the factors associated with scoliosis in Chaozhou city

Based on the previous screening results, we conducted a case-control study to explore the factors associated with IS. The sample size was calculated as follows: $n=\frac{{\left({Z}_{\propto}\sqrt{2\overline{p}\overline{q}}+{Z}_{\beta}\sqrt{p_0{q}_0+{p}_1{q}_1}\right)}^2}{{\left({p}_1-{p}_0\right)}^2}$, where *p*_0_ is the exposure rate of a related factor in the controls, and *p*_1_ is the exposure rate of a related factor in the cases, *q*_0_=1-*p*_0_, *q*_1_=1-*p*_1_. We selected the variable “Having myopia” to calculate the sample size, and the needed number is 150 for the case group and control group, respectively.

A self-designed questionnaire was addressed to collect the following information: demographic characteristics, postural habits, cognition of scoliosis, self-sensation of scoliosis with symptoms, physical condition, sleeping time, exercise time, and any other factors. The questionnaire was revised twice following a pre-survey and consulting spine specialists and epidemiologists. The participant students were requested to finish the questionnaire in about 30 min. If anyone could not understand any question, he/she would consult the trained teachers and investigators. After finished, the questionnaire was checked by the investigators at once, to avoid any questions being neglected.

### Statistical analysis

The database was established with EpiData (Jens M. Lauritsen, Odense, Denmark)with double entry and validation to ensure consistency testing. The IBM SPSS Statistic 26.0 (IBM Corp., Armonk, NY, USA) was used to statistical analyses. Demographic data were analyzed by descriptive statistical methods. The results were expressed as the percentage value or mean ± standard deviation. The continuous variables were performed by independent sample *t* tests, and the categorical variables were performed by chi-squared tests. Logistic regression analysis was performed to explore the factors associated with scoliosis. All the statistical tests were two-sided, and *P* < 0.05 was considered statistically significant.

## Results

### Demographics of the participants

Scoliosis screening was conducted on 5497 primary school students in Chaozhou city, including 2479 girls and 3018 boys (Table 1). In total, the positive rate of girls (8.71%) was 2.16 times as many as boys (4.04%). The prevalence rate increased with age, and the prevalence in girls is all higher than in boys in different age groups (*χ*^*2*^ = 51.45, *P* < 0.001, Table 1).

**Table 1:** The prevalence rates of idiopathic scoliosis among primary school children in Chaozhou, China

Age (years)	Male		Female	
	N	Positive, n (%)	N	Positive, n (%)
6	338	10(2.96)	308	20(6.49)
7	457	16(3.50)	400	27(6.75)
8	490	21(4.29)	388	33(8.51)
9	554	23(4.15)	471	34(7.22)
10	502	21(4.18)	428	31(7.24)
11	507	22(4.34)	379	41(10.82)
12	170	9(5.29)	105	30(28.57)
Total	3018	122 (4.04)	2479	216(8.71)

### 3D scanning on body surface

**Fig. 1:**
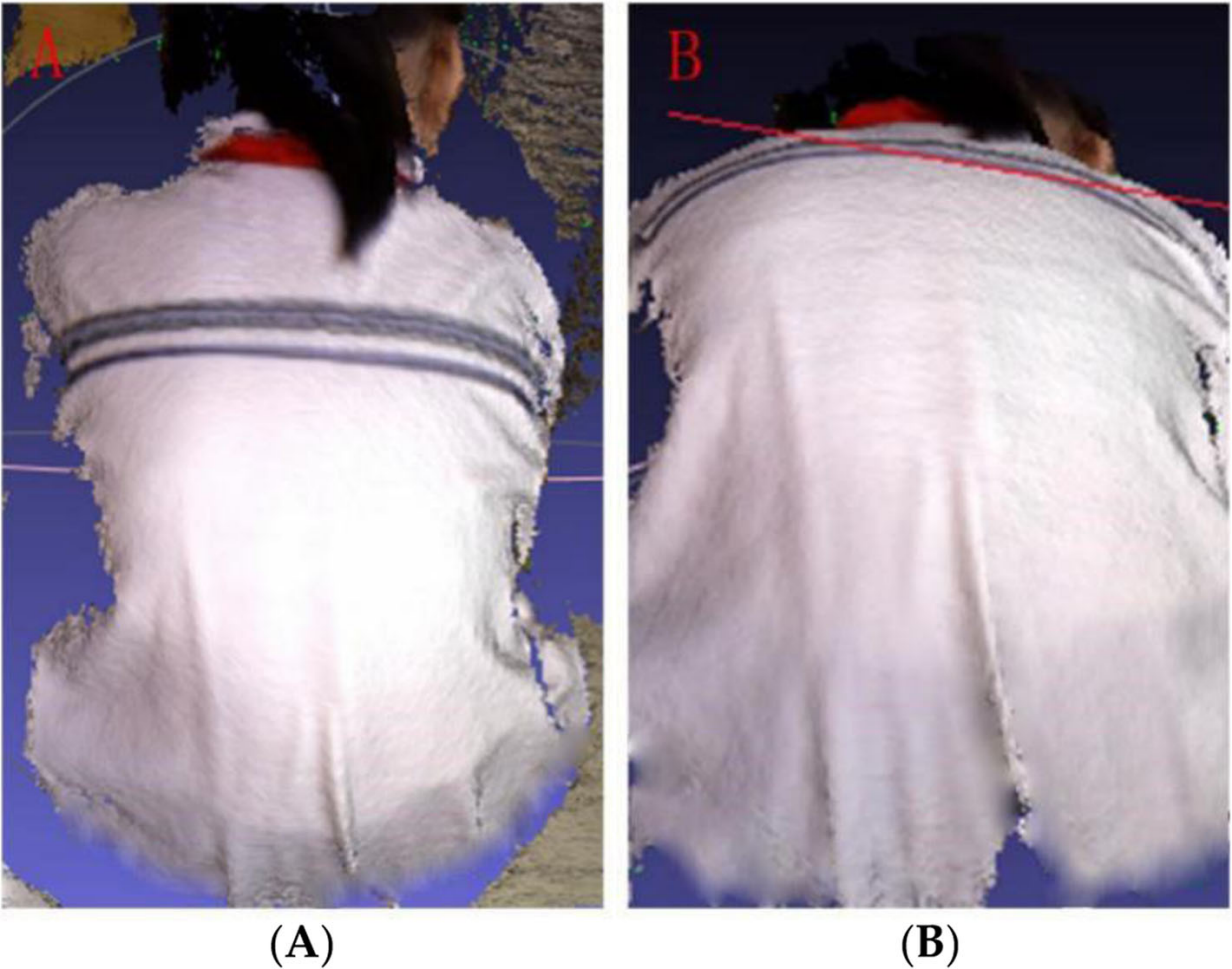
3D images of the back. **A** 3D image on the back. **B** An angle-adjusted 3D image. The line is the tangent of the plane of the chest and back, showing an obvious asymmetrical tilt of the chest and back

**Fig. 2:**
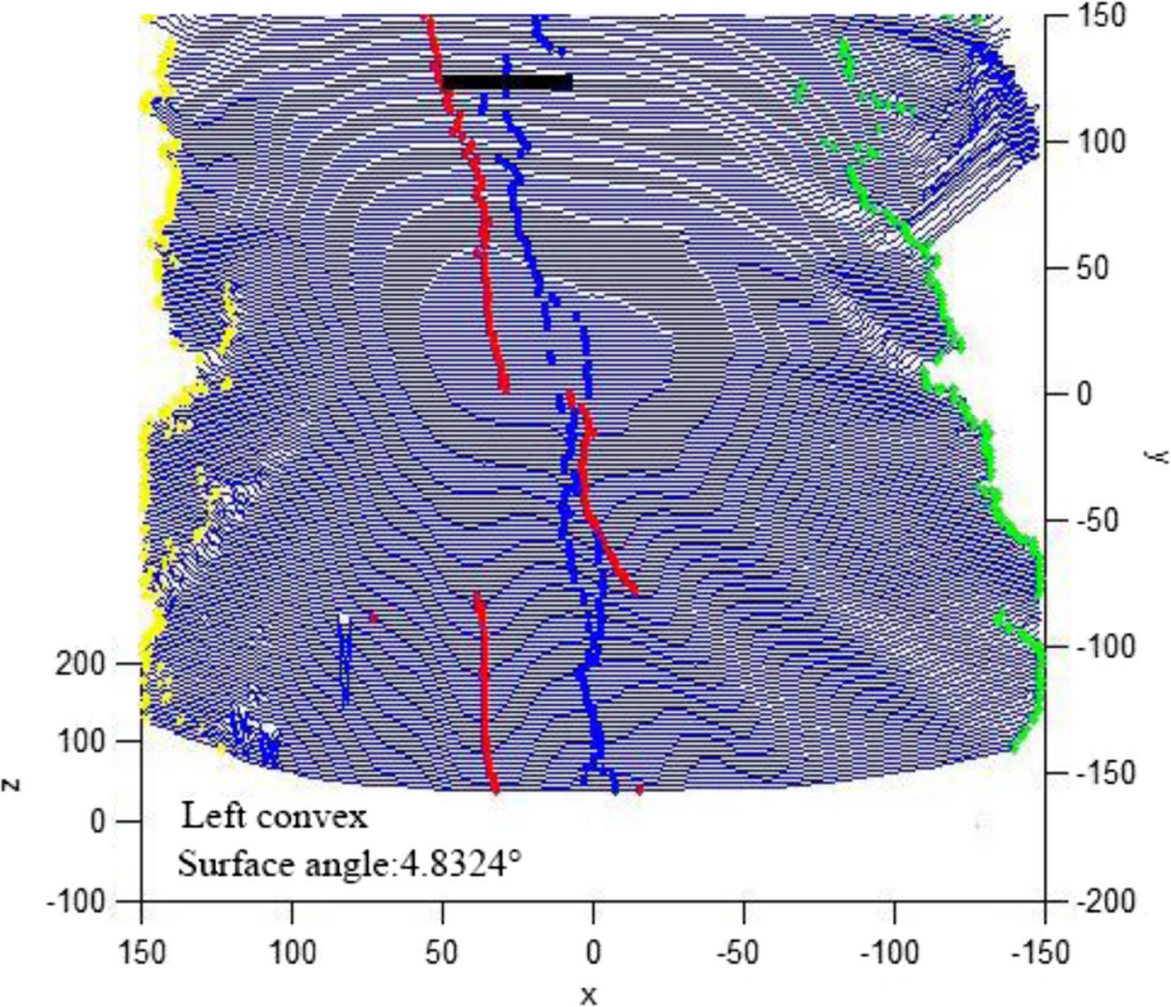
3D scanning image on body surface. The thick black line in the image is where the asymmetry of the back is most pronounced and where the inclination of the surface is measured

In this study, 3D scanning on the back of all positive pre-screened subjects (338) was recorded at MeshLab 3D. The 3D scanning was filtered to find the most obvious part of the back asymmetry and simulate the Scoliometer. The 3D images obtained in this screening showed that the minimum ART is 3°and the maximum ART is 8°. Figure 1 shows the 3D image on the back of a female scoliosis case, and the surface tilt angle is measured at 4.8324°(Fig. 2).

### ART of MeshLab 3D and Cobb angle of X-ray examination

A total of 12 screened positive patients participated in the reexamination, among which the minimum ART was 2.3° and the maximum was 10.4°. The minimum Cobb angle of X-ray examination was 8°, and the maximum was 37°. The ART of 11 patients was ≥3°, with a positive rate of 91.67%. The Cobb angle of 10 patients was ≥10°, with a positive rate of 83.33%, and the accuracy rate was 84.62% (Table 2).

**Table 2:** Comparison of ART and Cobb angle

		Cobb angle ≥ 10°	
ATR ≥ 3°		+	-
	+	10	1
	-	0	1

ATR ≥ 3 was screening value; Cobb angle ≥ 10° was confirming value

### Investigation on the factors associated with IS

A total of 2550 questionnaires were collected, among which 2547 (1094 girls and 1453 boys) were valid, with an effective response rate of 99.8%. Significant associations with IS were found in gender, weight, BMI, appetite, myopia, cognition of scoliosis, self-sensation of scoliosis with symptoms, sleep time, and exercise time (all *P* < 0.05, Table 3).

**Table 3:** Demographic characteristics of idiopathic scoliosis cases and controls

Variable	Cases (***n***=175)	Controls (***n***=2372)	***P***
Height (mean±SD, cm) Weight (mean±SD, kg)	142.64±12.89 32.74±8.28	141.64±12.78 34.35±10.32	0.323 0.045
BMI (mean±SD, kg/cm^2^)	16.03±3.22	16.91±3.95	0.004
Gender			
Female	116 (66.3)	978 (41.2)	<0.001
Male	59 (33.7)	1394 (58.8)	
Appetite			
Bad	3 (1.7)	111 (4.7)	0.034
General	117 (66.9)	1379 (58.1)	
Good	55 (31.4)	882 (37.2)	
Having myopia			
No	112 (64.0)	1774 (74.8)	0.002
Yes	63 (36.0)	598 (25.2)	
Have a habit of carrying a backpack on one shoulder			
Sometimes	54 (30.9)	758 (32.0)	0.215
No	110 (62.9)	1375 (58.0)	
Yes	11 (6.3)	238 (10.0)	
Cognition of scoliosis			
Never known	58 (33.1)	1231 (51.9)	<0.001
Have heard	62 (35.4)	687 (29.0)	
Known	55 (31.4)	453 (19.1)	
Self-sensation of scoliosis with symptoms			
No	65 (37.1)	1142 (48.2)	<0.001
Not clear	83 (47.4)	1136 (47.9)	
Yes	27 (15.4)	93 (3.9)	
Sleep time			
More than 10 h	2 (1.1)	117 (4.9)	0.049
8 to 10 h	156 (89.1)	1995 (84.1)	
Less than 8 h	17 (9.8)	260 (11.0)	
Daily exercise duration			
More than 3 h	1 (0.6)	117 (4.9)	0.036
2 to 3 h	10 (5.7)	175 (7.4)	
1 to 2 h	63 (36.2)	869 (36.7)	
Less than 1 h	100 (57.5)	1209 (51.0)	

Data are no. (%) unless indicated. Independent sample *t* test was used for the continuous variables, and chi-squared test or Fisher’s exact test was used for categorical data

Body coordination, vertebral discomfort, postural habits, and exercise methods between cases and controls were shown in Table 4. In terms of body coordination, 3.4% of the cases have the problem of asymmetrical back, 14.3% of the cases have hunchback, while 1.3% and 9.1%, respectively, in the controls (both *P*<0.05). In the survey of vertebral discomfort, 42.3% of the cases had neck pain, which was higher than the controls (33.2%, *P*=0.014). As to exercise methods, more IS cases engaged in badminton (54.3% vs. 44.9%), but less engaged in basketball (24.0% vs. 34.7%).

**Table 4:** Distribution of body coordination, vertebral discomfort, postural habits, and exercise adopted in daily activities in participants

Variables	Cases (***n***=175)	Controls (***n***=2372)	***P***
Body coordination			
Shoulder asymmetries	8 (4.6)	77 (3.2)	0.346
Unequal length of legs	1 (0.6)	41 (1.7)	0.361
Asymmetrical back	6 (3.4)	31 (1.3)	0.038
Hunchback	25 (14.3)	217 (9.1)	0.025
Crooked neck	8 (4.6)	60 (2.5)	0.137
Vertebral discomfort			
Neck pain	74 (42.3)	787 (33.2)	0.014
Chest pain	17 (9.7)	298 (12.6)	0.269
Waist pain	37 (21.1)	464 (19.6)	0.612
Postural habits			
Write or read on your stomach	32 (18.3)	442 (18.6)	0.909
Curl up with a book	15 (8.6)	162 (6.8)	0.382
Sleep on one side	109 (62.3)	1340 (56.5)	0.135
Paralyzed sitting	13 (7.4)	169 (7.1)	0.880
Sleep on your stomach	18 (10.3)	371 (15.6)	0.057
Cross your legs	20 (11.4)	295 (12.4)	0.696
Watch TV in bed	28 (16.0)	349 (14.7)	0.644
Play mobile phone in bed	60 (34.3)	947 (39.9)	0.141
Exercise methods			
Badminton	95 (54.3)	1065 (44.9)	0.016
Basketball	42 (24.0)	824 (34.7)	0.004
Football	20 (11.4)	269 (11.3)	0.972
Table tennis	20 (11.4)	313 (13.2)	0.503
Shuttlecock	7 (4.0)	87 (3.7)	0.822
Dance	31 (17.7)	331 (14.0)	0.169
Roller skating	43 (24.6)	536 (22.6)	0.548
Ride on a bicycle	132 (75.4)	1708 (72.0)	0.329
Chinese Kungfu	13 (7.4)	239 (10.1)	0.258
Running	102 (58.3)	1434 (60.5)	0.571
Swimming	63 (36.0)	877 (37.0)	0.797

Data are no. (%) unless indicated. Chi-squared test or Fisher’s exact test was used for categorical data

### Multifactorial analysis of correlative factors of IS

Univariate logistic regression analysis models were used to explore the factors associated with IS. The results showed that gender, myopia, cognition of scoliosis, self-sensation of scoliosis with symptoms, sleep time, and daily exercise duration were all related to the occurrence of IS (all *P*<0.05, Table 5).

**Table 5:** Univariate logistic regression analysis of the factors associated with idiopathic scoliosis

Variable	***P***	***OR***	95%***CI***
Gender			
Male		1.0	(Reference)
Female	<0.001	2.80	(2.03,3.87)
Having myopia			
No		1.0	(Reference)
Unclear	0.524	0.85	(0.52,1.40)
Yes	0.005	1.62	(1.16,2.26)
Keep good posture while reading			
No		1.0	(Reference)
Yes	0.947	1.01	(0.74,1.37)
Relax back after sitting for a long time			
Never		1.0	(Reference)
Sometimes	0.284	1.24	(0.84,1.83)
Usually	0.817	0.94	(0.55,1.60)
Self-sensation of scoliosis with symptoms			
No		1.0	(Reference)
Not clear	0.144	1.28	(0.92,1.79)
Yes	<0.001	5.101	(3.11,8.38)
Sleep time			
10 to 11 h		1.0	(Reference)
9 to 10 h	0.043	4.38	(1.05,18.35)
8 to 9 h	0.105	3.22	(0.78,13.26)
Less than 8 h	0.219	2.54	(0.57,11.28)
Duration of reading			
More than 3 h		1.0	(Reference)
2 to 3 h	0.820	0.92	(0.44,1.93)
1 to 2 h	0.503	0.79	(0.40,1.57)
Less than 1 h	0.929	0.97	(0.48,1.97)
Duration of exercise			
More than 3 h		1.0	(Reference)
2 to 3 h	0.072	6.69	(0.85,52.93)
1 to 2 h	0.035	8.48	(1.17,61.73)
Less than 1 h	0.025	9.68	(1.34,70.01)

*OR* odds ratio, *95% CI* confidence interval for *OR*

In the multiple logistic regression analysis model, females had more likelihood to develop IS (OR=2.45, 95%*CI* 1.71–3.36); myopia children presented 1.49 times (95%*CI* 1.05–2.11) higher likelihood of IS, when compared to children without myopia. With the BMI of the children increasing, the IS decreased (OR=0.67, 95%*CI* 0.23–0.89). The IS cases had more self-sensation of scoliosis symptoms (OR=5.52, 95%*CI* 3.27–9.29). The likelihood of scoliosis increased gradually as the sleep time decreased, and those who sleep less than 8 h were 3.33 times (95%*CI* 1.51–10.64) greater likelihood than those with more than 10 h a day. With the increase of exercise time, the likelihood of scoliosis decreased gradually, and those who exercised less than 1 h were 7.29 times (95%*CI* 1.99–53.37) greater likelihood than those with 3 h a day (Table 6).

**Table 6:** Multiple logistic regression analysis of the factors associated with idiopathic scoliosis

Variables	***P***	***OR***	95%***CI***
Gender (female vs. male)	<0.001	2.45	(1.71, 3.36)
BMI	0.032	0.67	(0.23, 0.89)
Having myopia			
No		1.0	(Reference)
Yes	0.026	1.49	(1.05,2.11)
Self-sensation of scoliosis			
No		1.0	(Reference)
Not clear	0.249	1.23	(0.87,1.74)
Yes	<0.001	5.52	(3.27,9.29)
Sleep time			
More than 10 h		1.0	(Reference)
8 to 10 h	0.039	2.65	(1.08,19.97)
Less than 8 h	0.014	3.33	(1.51,10.64)
Duration of exercise			
More than 3 h		1.0	(Reference)
2 to 3 h	0.103	5.39	(0.67,43.25)
1 to 2 h	0.045	7.09	(1.06,52.17)
Less than 1 h	0.025	7.29	(1.99,53.37)

*OR* odds ratio, *95% CI* confidence interval for *OR*

## Discussion

This study was conducted to survey the IS prevalence among primary school-aged children in Chaozhou, Southeast of China, and further investigate the factors associated with IS, which was the first time to screen IS in primary school students based on a large-scale epidemiological study. The results indicated that the prevalence of IS among primary school students in Chaozhou was 6.15%, with 4.04% in males and 8.71% in females. In addition, the prevalence of IS increased with age. The female students, with more possibility of trunk asymmetry, had a higher prevalence than the males. Many factors such as BMI, myopia, cognition of scoliosis, self-sensation of scoliosis with symptoms, sleep time, and exercise time were associated significantly with scoliosis.

### Gender, age-related prevalence

Our study indicated that females had more possibility to suffer from IS. As reported in literature elsewhere, IS was more common in females. Adolescent girls are easier to suffer from idiopathic scoliosis because the curvature of the spine progress in puberty, and females enter physiological puberty early. Another reason is that boys are more motivated than girls when it comes to doing physical exercise [17], except in relation to weight and body image and agility or flexibility, which is superior in the female gender. The reduced body weight, especially in girls, strongly predisposes the occurrence of scoliosis. In the annual observation, the girls who had trunk asymmetry, in order to decrease the ATR value, cannot significantly increase their body weight [18]. The studies [19, 20] showed that adolescents with scoliosis were taller than other adolescents in the same age group, and a growth velocity of more than 2 cm per year was associated with curve progression. It is believed that the increased prevalence of scoliosis in girls compared to boys is justified by the fact that girls tend to grow more than boys from ages 11 to 13 [21]. The ratio of females to males was reported to range from 2.3 to 18 [22–24]. The previous studies [25, 26] reported that females had a higher prevalence in all age groups than that in males, and both genders had a higher prevalence in the age group 15–16 after puberty, which is consistent with our study.

### Weight, BMI, and IS

Weight is one of the important indexes to reflect a children’s health condition. The average weight of the IS cases is 32.74±8.28 kg, which is less than that of the controls (34.35±10.32 kg). At the developmental stage of a child, weight can reflect the nutritional status, muscle development, and bone development to some degree. BMI was a more comprehensive index of reflecting the body shape. The BMI of the IS cases is 16.03±3.22 kg/m^2^, which is also less than that of the controls (16.91 ±3.95 kg/m^2^), but both fall within the normal range of the national standard for students’ physical health. The study [27] indicated a population-based prospective study, and the results suggested that BMI/body weight at age 10 and scoliosis at age 15 had a negative association, with per SD increase in BMI 20% reduced risk of scoliosis. Worthington et al .[28] indicated that malnutrition might play a crucial part in the etiology of IS. But nutrition covers a wide range of areas that still need to be studied in conjunction with other indicators.

### Myopia and IS

Students with myopia presented 1.49 times more possibility of IS, when compared with students who did not have myopia. It can be considered as a process of mutual or simultaneous development. Incorrect posture will increase the likelihood of scoliosis and lead to impaired vision. The major cardinal feature is joint hypermobility, or ligamentous laxity, [29, 30]. In the absence of vision rectification, the impaired vision will promote the creation of forced posture, which in turn exacerbated the occurrence of scoliosis [31]. This is in line with Egorova’s [32] study, which found that the musculoskeletal system of high myopia and impaired vision school-age children was more distorted than the controls, including scoliosis, pelvic dislocation, kyphosis, lordosis, torsion of the column, flatfoot, lower limb, and chest deformation.

### Cognition and self-sensation of scoliosis

The IS cases had higher self-sensation of scoliosis symptoms. The most common scoliosis symptoms are physical asymmetry, back pain injury, muscle spasms, and others. According to the studies [33, 34], idiopathic scoliosis may result in a difference in leg length which regards that the difference in leg length can lead to compensated non-progressive lumbar scoliosis. In the published study [35], smaller leg length differences (≤ 2 cm) may lead to functional or non-fixed scoliosis, which should not be overlooked by medical professionals. Postural imbalances caused by muscle spasms, injury, pain, or any other factor s[36] can also develop into unstructured scoliosis, which is generally considered to be inconsequential [37]. However, if functional scoliosis is not diagnosed and corrected in puberty, it may eventually develop into pathological scoliosis [38]. Even if the screening results were normal, students with physical asymmetry still need to be paid great attention to, especially in puberty.

The cognition and sensation of deformity with IS should be considered cautiously, for the best treatment methods can be applied to reduce the spine curvature or stop the progression of IS [39, 40]. Having good cognition help students redress their bad habits and took corrective measures initiatively. In theory, good posture can reduce the asymmetrical load of column deformity and reverse the vicious cycle of column curvature. Incorrect postures are common in daily life, which will increase the load of column asymmetry [41].

### Sleep time, exercise time, and IS

Scoliosis patients have an unbalanced load that may lead to skeletal muscle fatigue and require more sleep to regain strength [42]. From another perspective, most muscles are relaxed when people are lying. The most obvious change after standing up is the erector spine, which is an important muscle for keeping the body upright and maintaining the stability and balance of the crista e[43]. Long time lying may lead to poor vertical stress training and muscle strength or endurance of skeletal muscl e[44]. The study [45] showed that decreasing sleep duration results in overexpression of IL-1 and lower bone mineral density and might increase the spine curvature. Furthermore, the relationship of scoliosis, class II malocclusion, and obstructive sleep apnea is worth discussing. The study [46] showed that the forward head posture associated with scoliosis may lead to stretching of the muscles, skin, and fascia covering the head and neck, impeding the sagittal growth of the mandible and facial skeleton, which leads to class II malocclusion. Besides, class II malocclusion has been reported as a dental feature associated with obstructive sleep apnea which could explain sleeping disturbances in IS patients [47]. The stud y[48] suggested that sleep screening should be taken into consideration for the evaluation and treatment of patients with early-onset scoliosis, because poor-quality and inadequate sleep has a bad influence on children’ behavior, cognitive function, and growth.

With the increase of exercise time, the likelihood of scoliosis decreased gradually, and those who exercised less than 1 h were 7.29 times more likelihood than those with 3 h a day. Since it is found that biomechanics are relative to unbalanced loads and time thresholds, we can reverse the asymmetrical load by restoring normal postures and exercise. According to the study [49], exercise can reduce the prevalence of scoliosis and patients Cobb angle, especially in early adolescence, because exercise can improve mobility, strength, breathing, and equilibrium. The study [50] confirmed the efficacy of exercises can reduce spine curvature (mainly in puberty) and improve the Cobb angle. The study [51, 52] showed the effectiveness of spinal stabilization exercises on pain reductions for adolescents with idiopathic scoliosis.

### Limitation and strength

This study has several limitations. Firstly, X-ray examination was not performed immediately because of low compatibility and lack of attention. Secondly, the cross-sectional study could not establish the cause-and-effect relationship, while a longitudinal approach might help research the development of the relationship. Third, the number of participants in the case-control study was reduced because it was conducted seven months after screening. Despite these limitations, FBT and ATR of 3D images used in the survey could help us increase the accuracy and objectivity of the screening. More adolescents and age phases are needed to be investigated in the future based on large-scale epidemiological studies to reach more scientific conclusions. To our knowledge, this is the first study conducted in Chaozhou city to screen and investigate influencing factors of IS in primary school children. In the future, a wider group of predictors and broader age groups should be brought into study, to collect more epidemiological data and more clues for the scoliosis intervention.

## Conclusions

Scoliosis is still common in primary school students due to multiple influencing factors. IS prevalence among female students in Chaozhou city is significantly higher than that of males. Besides, lower body mass, having myopia, insufficient sleep time, and lower physical activity are associated with IS. Since the prevalence of IS increases with age, early screening should be carried out before puberty, especially in adolescent females.

## Data Availability

The datasets used and/or analyzed during the current study are available from the corresponding author on reasonable request.

## Abbreviations

IS: Idiopathic scoliosis
FBT: Adams’ forward bending test
ATR: Angle of trunk rotation
BMI: Body mass index
